# P126: Direct observation survey of practice of alcohol-based handrubbing in Fann Teaching Hospital, Dakar, Senegal

**DOI:** 10.1186/2047-2994-2-S1-P126

**Published:** 2013-06-20

**Authors:** BA Niang, MN Chraiti, S Bagheri Nejad, NM Dia, ML Diouf, NA Diop, J Hightower, MB Diallo, M Seydi

**Affiliations:** 1 Infectious Diseases department, FANN teaching hospital, Dakar, Senegal; 2 Service of control and prevention, HUG, Geneva, Switzerland; 3 WHO, Geneva, Switzerland

## Introduction

Health care-associated infections (HCAI) result essentially from cross-transmission of pathogenic microorganisms by the hands of healthcare workers (HCW). Their care represents a universal challenge in practice.

## Objectives

Our study aimed to measure HCW compliance with hand hygiene.

## Methods

We conducted a direct observance of hand hygiene compliance of HCW over a period of three months, based on the WHO’s “five indications of the hand hygiene” approach.

## Results

For a total of 338 opportunities, the rate of global observance of hand hygiene was 36.1% with 80.3% of handrubbing realized. According to the department, this rate of observance was variable: Pneumology (42.3%), Thoracic Surgery and Cardiovascular (58.6%), Neurology (20%), Neurosurgery (24%), Emergency (25%), Laboratories (30%), Infectious diseases (39%), Psychiatry (33.3%), ORL (25%), Oral department (44.4%). According to the professional category, the observance was the following one: doctors (50.6%), nurses (34%) auxiliaries (29.1%) other nursing staffs (43.8%). The level of use of Alcohol-based handrub (ABHR) during hand hygiene was: auxiliaries (93%), doctors (82.1%), nurses (75.8%), others (14.3%). The observance of ABHR according to "five indications" was 87.7% before patient contact, of 83.3% before aseptic procedure, 44.4% after a risk of body fluid exposure, of 78% after patient contact and of 100% to the immediate surroundings of patient.

## Conclusion

Observance of hand hygiene with ABHR is still low in the structure. A training program coupled with a sharing experience of outcomes of the survey should allow to improve it.

## Disclosure of interest

None declared

